# Daytime and nighttime casein supplements similarly increase muscle size and strength in response to resistance training earlier in the day: a preliminary investigation

**DOI:** 10.1186/s12970-018-0228-9

**Published:** 2018-05-15

**Authors:** Jordan M. Joy, Roxanne M. Vogel, K. Shane Broughton, Urszula Kudla, Nathaniel Y. Kerr, Jason M. Davison, Robert E. C. Wildman, Nancy M. DiMarco

**Affiliations:** 1 Nutrition and Food Sciences Department, Texas Woman’s University, 304 Administration Drive, Denton, TX 76204 USA; 2 Institute for Women’s Health, Texas Woman’s University, 013 Human Development Building, P.O. Box 425876, Denton, TX 76204-5888 USA; 3 GU Energy Labs, Berkeley, CA 94710 USA; 4 Friesland Campina, 6700 AE Wageningen, Amersfoort, The Netherlands; 5 Dymatize Athletic Nutrition Institute, Dallas, TX 75207 USA

**Keywords:** Protein, Timing, Hypertrophy, Supplements, Resistance training, Window of opportunity, Anabolic window

## Abstract

**Background:**

Casein protein consumed before sleep has been suggested to offer an overnight supply of exogenous amino acids for anabolic processes. The purpose of this study was to compare supplemental casein consumed earlier in the day (DayTime, DT) versus shortly before bed (NightTime, NT) on body composition, strength, and muscle hypertrophy in response to supervised resistance training.

**Methods:**

Thirteen males participated in a 10-week exercise and dietary intervention while receiving 35 g casein daily. Isocaloric diets provided 1.8 g protein/kg body weight.

**Results:**

Both groups increased (*p* < 0.05) in lean soft tissue (DT Pre: 58.3 ± 10.3 kg; DT Post: 61.1 ± 11.1 kg; NT Pre: 58.3 ± 8.6 kg; NT Post: 60.3 ± 8.2 kg), cross-sectional area (CSA, DT Pre: 3.4 ± 1.5 cm2; DT Post: 4.1 ± 1.7 cm2; NT Pre: 3.3 ± 1.6 cm2; NT Post: 3.7 ± 1.6 cm2) and strength in the leg press (DT Pre: 341 ± 87.3 kg; DT Post: 421.1 ± 94.0 kg; NT Pre: 450.0 ± 180.3 kg; NT Post: 533.9 ± 155.4 kg) and bench press (DT Pre: 89.0 ± 27.0 kg; DT Post: 101.0 ± 24.0 kg; NT Pre 100.8 ± 32.4 kg; NT Post: 109.1 ± 30.4 kg) with no difference between groups in any variable (*p* > 0.05).

**Conclusions:**

Both NT and DT protein consumption as part of a 24-h nutrition approach are effective for increasing strength and hypertrophy. The results support the strategy of achieving specific daily protein levels versus specific timing of protein ingestion for increasing muscle mass and performance.

**Trial registration:**

ClinicalTrials.gov Identifier: NCT03352583.

## Background

Dietary protein optimizes resistance training adaptations for muscle mass accretion [1–3]. Greater protein requirements for athletes and active individuals training to build muscle, combined with complex schedules and/or lifestyles may compromise nutrition strategies that enhance muscle hypertrophy over time. Total daily intake of protein appears to be the most potent factor in maximizing muscle adaptation in conjunction with resistance training [4]. However, the concept of protein timing implies the peri-workout and pre-sleep time periods may have a special role in optimizing dietary proteins for athletic purposes [5, 6].

The potential role of nighttime nutrition in muscle adaptation to training is often overlooked. However, during sleep, ingested protein is digested and absorbed equivalent to non-sleeping periods [7, 8]. Muscle and other tissues respond to hyperaminoacidemia during sleep by increasing muscle protein synthesis (MPS) when prior resistance exercise occurs in the evening [7, 8]. In a 12-week study, the combination of evening resistance training and supplemental casein protein lead to greater gains in strength and muscle mass than resistance training alone [6]. While the resistance training stimulus was consistent across all participants, the group receiving the supplemental casein had a significantly greater daily protein intake (1.9 vs. 1.3 g • kg^− 1^ • d^− 1^), as the control group did not receive an isocaloric or isonitrogenous comparator. Furthermore, since the training stimulus was during the evening, followed by supplemental protein, influential elements of protein timing might exist. Thus, one of the purposes of this study was to determine whether increased protein intake would be equally effective if the protein was consumed before bed, versus earlier in the day and closer to an earlier training stimulus.

Athletes are often hesitant to eat late into the evening due to the perception that it would disrupt body fat breakdown during sleep and, in turn, leanness. However, single night studies involving supplemental protein prior to bed suggest that this action may not significantly disturb lipolysis and fat oxidation overnight [9–11]. Since protein consumption closer to sleep likely does not influence body leanness, such a practice can help with daily planning and achieving appropriate levels of dietary protein. Limited research has been performed examining exercise training and supplemental protein on potential changes in muscle size and performance and adipose tissue simultaneously. In one study, males and females already engaged in unsupervised exercise training were provided 54 g of casein protein, either at night or in the morning, for 8 weeks [12]. Protein intake was increased in both groups from 1.7–1.8 to 2.4 g • kg^− 1^ • d^− 1^ and no differences in strength and body composition from beginning to end were observed. Yet to date, an incremental, high-intensity, monitored training program has not been conducted in conjunction with supplemental casein protein (night vs day) on measures of muscle thickness, body composition and strength. Thus to our knowledge, this is the first longitudinal isonitrogenous, isocaloric, nighttime casein supplementation study investigating the impact on body weight (BW) and composition as well as strength and muscle hypertrophy when an impactful resistance training stimulus occurs earlier in the day. It was hypothesized that the nighttime (NT) supplemented group would experience greater benefit to resistance-training induced physiological changes. The results of this study are important to athletes and active individuals who train for performance, aesthetics, and health.

### Methods

#### Experimental design

In a randomized, double-blind, placebo-, diet-, and exercise-controlled trial, participants in the NT group were supplemented with 35 g casein protein at night immediately before going to sleep and 35 g maltodextrin earlier in the day, and participants in the daytime group (DT) were supplemented with 35 g maltodextrin at night immediately before going to sleep and 35 g casein protein earlier in the day. Participants were randomized to the NT or DT group by stratified randomization based on cross-sectional area of the rectus femoris (CSA) to balance groups based on muscle size and strength. The supplement taken early in the day was not consumed within 3 h of beginning or ending exercise, nor was it consumed within 6 h of sleep. Exercise programs and diets were prescribed for each participant, and both were supervised and tracked throughout the intervention. Prior to and following the intervention period, participants’ body composition, muscle hypertrophy, and athletic performance were assessed.

#### Participants

Healthy, recreationally active, 18–25-year-old males (NT: 71.4 ± 11.1 kg, 170.2 ± 3.8 cm training 4.0 ± 0.9 days/week for prior 2.7 ± 0.52 years; DT: 79.5 ± 21.5 kg, 178.1 ± 6.5 cm training 3.7 ± 1.1 days/week for prior 2.0 ± 0.82 years) were screened for participation. Individuals were eligible if they engaged in regular exercise for the previous 1–3 years at a frequency of 2–5 days per week and were excluded for tobacco use, excessive alcohol intake (≥ 12 drinks/week), having history of medical or metabolic complications, as well as use of nutritional supplements or medications that would significantly affect study outcomes. Study protocols were approved by the Institutional Review Board at Texas Woman’s University, and informed consent was provided by all participants prior to the investigation.

#### Measurements

BW was assessed using a physician’s scale (BWB-800, Tanita Corporation, Tokyo, Japan) and height by stadiometer. Dual-Energy X-ray Absorptiometry (DXA; Lunar Prodigy, General Electric Corporation, Boston, MA) was used to assess fat mass (FM), body fat percentage (BF%), lean soft tissue (LST), and appendicular LST (ALST). Test–retest reliability for DEXA measurements in15 subjects, resulted in an average intraclass correlation (ICC) of > 0.99. Ultrasonography-determined (Logiq e, General Electric Corporation, Boston, MA) CSA of the rectus femoris and combined muscle thickness (MT) of the vastus lateralis and vastus intermedius were measured as previously described [13]. Briefly, CSA measurements were conducted on the anterior thigh at 75% femur length, defined as the distance from the anterior superior iliac spine to the superior aspect of the patella, and MT measurements were conducted at 50% femur length, defined as the distance from the greater trochanter to the lateral epicondyle of the femur. Test–retest reliability for ultrasound measurements, as determined using 5 subjects, resulted in an average ICC > 0.99. All body composition measurements were conducted in the morning following an overnight fast while the participant wore only lightweight athletic shorts, a t-shirt, and socks. Leg press and bench press 1-repetition-maximum (1RM) testing determined changes in strength. Participants were required to allow the sled to descend to a knee angle of 90^0^ and press back to the starting position for a successful attempt in the leg press 1RM. In the bench press 1RM, they were required to touch the bar to their chest without bouncing and press back to the starting position without lifting their hips from the bench. A standard 3-min or 5-min rest period was used between all warm up sets or 1RM attempts, respectively. Participants first warmed up with the bar, followed by an initial weight equal to approximately 50% of 1RM and increased intensity progressively over 3–4 sets up to ~ 85% 1RM for a single repetition prior to beginning 1RM attempts at ~ 90% estimated 1RM. During mid and post 1RM testing, the final warm up set was performed at an intensity 2.3 kg less than their previously determined 1RM for one repetition, and participants first 1RM attempt was performed at an intensity 2.3 kg more than their previously determined 1RM. Thereafter, intensity was increased by 2.3–22.7 kg per attempt according to participants’ apparent capabilities observed by qualified researchers. Vertical jump (VJ) testing was conducted to assess changes in jump height (Vertec, Perform Better, Cranston, RI), during which peak power (PP) and velocity (PV), average power (AP) and velocity (AV), and peak force (PF) were determined using a linear force transducer (Weightlifting Analyzer, Tendo Sports Machines, Slovak Republic) fastened to the back of a thin canvas belt tightened over top participants’ waistbands during their jump attempt. Participants were weighed fully clothed for accurate loads to be entered into the force transducer. Test-retest reliability for performance measures, as determined using 4 participants, resulted in an average ICC > 0.96. All pre and post testing measures were conducted at the same time of day to prevent diurnal variations. Delayed onset muscle soreness (DOMS) and rating of perceived exertion (RPE) were measured at the beginning and end of each exercise session, respectively, using a 10 cm visual analogue scale.

#### Resistance training protocol

Exercise sessions took place at training facilities on campus and were monitored by National Strength & Conditioning Association Certified Strength & Conditioning Specialists. The exercise stimulus was a periodized resistance training intervention consisting of two 5-week mesocycles, which trained each major muscle group twice weekly. Within each mesocycle, intensity increased as repetitions decreased (Table 1). During week 5, the upper and lower body strength oriented training sessions began with 1RM testing to more accurately prescribe training loads during the following mesocycle. During week 10, the Thursday and Friday sessions were composed of only a warm-up to 1 repetition with each participant’s 1RM from week 5. Intensity and number of sets and repetitions were recorded during every training session.

**Table 1:** Resistance Training Schedule

Weeks 1–5							
Monday		Tuesday		Thursday		Friday	
Lower Hypertrophy		Upper Hypertrophy		Lower Strength		Upper Strength	
Leg Press	5 × 6–15	Bench Press	5 × 6–15	Leg Press	5 × 1–5	Bench Press	5 × 1–5
SS Box Squat	4 × 6–15	Decline Press	3 × 6–15	Hack Squat	3 × 1–5	DB Press	3 × 3–8
Hyperextension	3 × 6–15	Incline Flye	3 × 6–15	Lunge	3 × 3–8	Shoulder Press	3 × 3–8
1-Leg Extension	3 × 6–15	Machine Shoulder Press	3 × 6–15	1-Leg Extension	3 × 6–15	Chest Supported Row	3 × 3–8
2-Leg Curl	3 × 6–15	Lateral Raise	3 × 6–15	2-Leg Curl	3 × 6–15	Pulldown	3 × 3–8
Calf Press	3 × 6–15	Low Cable Row	5 × 6–15	Leg Raise	3 × 10–20	YTWL	3 × 6
1-Tricep Extension	3 × 6–15	Pulldown	3 × 6–15				
2-Bicep Curl	3 × 6–15	1-Cable High Row	3 × 6–15				
Cable Abdominal Crunch	3 × 10–20	2-Rear Delt Flye	3 × 6–15				
Weeks 6–10							
Monday		Tuesday		Thursday		Friday	
Lower Hypertrophy		Upper Hypertrophy		Lower Strength		Upper Strength	
Leg Press	5 × 6–15	Bench Press	5 × 6–15	Leg Press	5 × 1–5	Bench Press	5 × 1–5
Front Squat	4 × 6–15	Incline Press	3 × 6–15	SS Box Squat	3 × 1–5	Pause Press	3 × 1–5
V-Squat Good Morning	3 × 6–15	Decline DB Press	3 × 6–15	DB Lunge	3 × 3–8	DB Shoulder Press	3 × 3–8
1-Leg Extension	3 × 6–15	Shoulder Press	3 × 6–15	1-Leg Extension	3 × 6–15	Cable Low Row	3 × 3–8
2-Leg Curl	3 × 6–15	Cable+DB Lateral Raise	3 × 6–15	2-Leg Curl	3 × 6–15	Pulldown	3 × 3–8
Calf Press	3 × 6–15	Chest Supported Row	5 × 6–15	Leg Raise	3 × 10–20	YTWL	3 × 6
1-Supine Tricep Extension	3 × 6–15	Pulldown	3 × 6–15				
2-Preacher Curl	3 × 6–15	1-Cable High Row	3 × 6–15				
Abdominal Crunch	3 × 10–20	2-Rear Delt Flye	3 × 6–15				

Exercise selection and order for each mesocycle is listed as well as the sets and repetitions for each exercise (SETSxREPS). Rest periods were 90 s for hypertrophy sessions and 3–5 min for strength sessions. “SS” indicates use of a safety squat bar, “DB” indicates dumbbell, and “YTWL” is a shoulder mobility exercise principally involving shoulder flexion and scapular retraction and depression movements. 1-exercise, 2-exercise indicate use of a superset. All participants performed the same number of sets and repetitions (±1 repetition per set) for each exercise. Individual capabilities were met by manipulating intensities for each exercise so the desired number of repetitions could be completed and participants would approach or achieve muscular failure by the final set. Each 5-week mesocycle began at the greatest number of repetitions and decreased week-by-week by 1–3 repetitions to the least number of repetitions indicated (e.g., for a range of 6–15 repetitions, week 1 = 15 repetitions, week 2 = 12 repetitions, week 3 = 10 repetitions, week 4 = 8 repetitions, week 5 = 6 repetitions). Concurrently and proportionally, intensity was increased as the number of repetitions decreased. Bench press and leg press intensities were prescribed as a percentage of 1RM, in which 5 repetitions were performed at a load of 85% 1RM, and load was increased or decreased by 2.5% 1RM for every 1 repetition decrease or increase, respectively

### Diet and protein supplementation

Caloric targets were established by Mifflin St. Jeor equation with a 1.6× adjustment for activity with 1.8 g protein/kg BW inclusive of the casein supplement, and the remainder of calories were provided as 35% fat and 65% carbohydrate. Total daily energy intake ratios corresponded to 20% protein, 52% carbohydrate, and 28% fat. Dietary compliance was monitored throughout the study by weekly intake surveys recorded using commercially-available software (MyFitnessPal, Baltimore, Maryland). Participants met weekly with researchers to assist them in reaching their dietary goals. Twenty-five grams of whey protein (ISO100, Dymatize, Dallas, TX) was provided to all participants post workout. Casein (as calcium caseinate; Friesland Campina, Amersfoort, The Netherlands) and maltodextrin supplements were flavor- and color-matched by the research staff. Each serving of casein provided 35 g of protein, < 0.5 g of fat, and < 0.5 g carbohydrate (lactose), and each serving of maltodextrin provided 35 g of carbohydrate, < 0.5 g fat, and < 0.5 g protein. Participants were provided canisters of casein and placebo at weeks 0, 3, and 6 with a supplement log to record time of consumption each day. Canisters were weighed before and after being given to the participants as a confirmatory measure of compliance.

### Statistical analysis

Data were analyzed using repeated measures ANOVA with Bonferroni post-hoc. Analyses were performed using Statistica software (Version 10, Dell, Round Rock, TX) and presented as means ± standard deviations. The alpha was set at *p* < 0.05.

## Results

### Participants

Twenty males began the study and 13 completed the entire 10 weeks while being at least 80% compliant with the diet and exercise intervention. Four participants were removed from analyses for maintaining less than 80% compliance per their reported diet logs or training session attendance. One participant from each group was removed from analyses for unreported noncompliance as validated by negative changes in both FM and LST and by participant interview after the study. One individual was removed from analyses for being an outlier for FM and body weight (> 3 SDs from mean). There was no significant (*p* > 0.05) difference between groups in any variables at baseline.

### Nutritional intake

Total and non-supplemented intake levels for calories, protein, carbohydrate and fat are provided in Table 2. Calories and macronutrient intakes did not differ (*p* > 0.05) between groups.

**Table 2:** Average Nutritional Intake

Measure	Unit	Group	Daily	Total Energy (%)	Units/Kg BW
Total Energy (Calories)	Cal	NT	2840.3 ± 192.5	100 ± 0.0	40.3 ± 4.5
		DT	3041.4 ± 343.3	100 ± 0.0	39.4 ± 5.0
Food Energy (Calories)	Cal	NT	2520.3 ± 192.5	88.8 ± 0.45	35.7 ± 3.9
		DT	2721.4 ± 343.3	89.3 ± 1.30	35.1 ± 4.1
Total Protein	g	NT	153.0 ± 26.5	21.5 ± 2.9	2.1 ± 0.2
		DT	158.0 ± 29.5	20.7 ± 1.5	2.0 ± 0.2
Food Protein	g	NT	118.0 ± 26.5	17.8 ± 3.5	1.6 ± 0.2
		DT	123.0 ± 29.5	17.2 ± 2.0	1.5 ± 0.1
Total Carb	g	NT	357.6 ± 29.4	50.4 ± 2.6	5.1 ± 0.8
		DT	392.6 ± 35.2	51.8 ± 3.2	5.1 ± 0.9
Food Carb	g	NT	322.6 ± 29.4	50.4 ± 3.0	4.5 ± 0.7
		DT	357.6 ± 32.5	52.0 ± 3.8	4.6 ± 0.8
Total Fat	g	NT	85.8 ± 5.9	27.3 ± 2.1	1.2 ± 0.2
		DT	96.2 ± 14.5	28.4 ± 2.4	1.2 ± 0.2
Food Fat	g	NT	85.8 ± 5.9	30.7 ± 2.6	1.2 ± 0.2
		DT	96.2 ± 14.5	31.8 ± 2.7	1.2 ± 0.2

Rows reading “Total” represent dietary and supplemental nutrition, and rows reading “Food” represent only dietary intakes without treatment supplements

### Anthropometrics and body composition

**Fig. 1:**
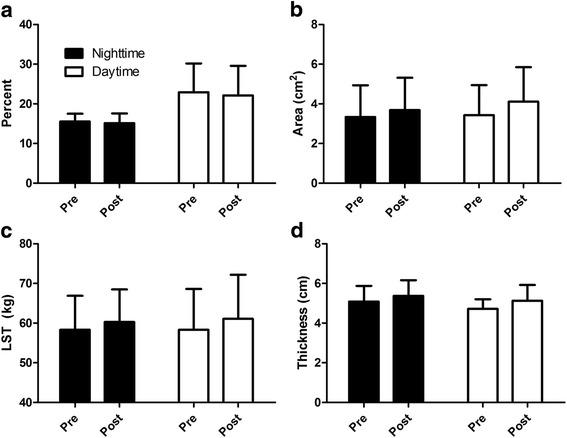
Changes in Body Composition and Muscle Hypertrophy. **a** BF%; **b** CSA; **c** LST; **d** MT. All variables presented in Fig. 1 had a main effect for time (*p* < 0.05). Data are presented as Mean ± SD

A significant (*p* < 0.05) main effect for time was observed for BW, LST, ALST, BF%, CSA, and MT (Table 3). BW, LST, ALST, CSA, and MT increased over time, while BF% decreased over time. No other main effects or interactions were observed for body composition variables, indicating the change in BF% was driven by changes in LST (Fig. 1).

**Table 3:** Changes in Anthropometric Measures

Measure	Unit	Group	Week 0	Week 12	Change
Body Weight^a^	kg	NT	71.4 ± 11.1	73.2 ± 11.0	1.8 ± 1.6
		DT	79.5 ± 21.5	83.0 ± 22.7	3.4 ± 1.4
Fat Mass	kg	NT	10.9 ± 2.7	10.9 ± 2.9	0.0 ± 0.8
		DT	18.7 ± 11.8	18.7 ± 12.0	0.0 ± 0.6
Body Fat Percentage^a^	%	NT	15.6 ± 1.9	15.2 ± 2.4	−0.4 ± 1.1
		DT	22.9 ± 7.2	22.1 ± 7.4	−0.8 ± 0.8
Lean Soft Tissue^a^	kg	NT	58.3 ± 8.6	60.3 ± 8.2	2.0 ± 1.5
		DT	58.3 ± 10.3	61.1 ± 11.2	2.8 ± 1.1
Appendicular LST^a^	kg	NT	27.1 ± 4.1	28.3 ± 3.7	1.2 ± 0.8
		DT	26.6 ± 5.0	28.2 ± 5.2	1.5 ± 0.4
Cross Sectional Area^a^	cm^2^	NT	3.3 ± 1.6	3.7 ± 1.6	0.4 ± 0.2
		DT	3.4 ± 1.5	4.1 ± 1.7	0.7 ± 0.5
Muscle Thickness^a^	cm	NT	5.1 ± 0.8	5.4 ± 0.8	0.3 ± 0.2
		DT	4.7 ± 0.5	5.1 ± 0.8	0.4 ± 0.5

^a^indicates a main effect for time

### Performance and perceptual measures

No significant differences (*p* > 0.05) were found for total training volume between groups (NT: 414,478.5 ± 98,618.0; DT: 381,397.0 ± 83,108.3 kg). A significant main effect for time (*p* < 0.05) was observed for leg press and bench press 1RM. A significant group x time interaction (p < 0.05) was observed for vertical jump peak force. No other significant (*p* > 0.05) effects or interactions were observed for performance measures (Table 4).

**Table 4:** Performance Measures

Measure	Unit	Group	Week 0	Week 12	Change
Leg Press 1RM^a^	kg	NT	450.0 ± 180.3	533.9 ± 155.4	83.9 ± 34.7
		DT	340.9 ± 87.3	421.1 ± 94.0	80.2 ± 35.7
Leg Press 1RM^a^	kg/kg BW	NT	100.8 ± 32.4	109.1 ± 30.4	8.3 ± 5.3
		DT	89.0 ± 27.0	101.0 ± 24.0	12.0 ± 5.4
Bench Press 1RM^a^	kg	NT	6.1 ± 1.8	7.4 ± 1.3	1.2 ± 0.7
		DT	4.3 ± 0.85	5.4 ± 1.2	1.1 ± 0.5
Bench Press 1RM^a^	kg/kg BW	NT	1.4 ± 0.2	1.5 ± 0.2	0.1 ± 0.1
		DT	1.1 ± 0.2	1.3 ± 0.2	0.2 ± 0.1
Vertical Jump Height	cm	NT	67.1 ± 4.8	69.4 ± 5.2	2.3 ± 5.8
		DT	55.2 ± 6.3	56.2 ± 7.6	1.1 ± 3.0
Vertical Jump PP	W	NT	8869 ± 3428	9040 ± 2983	171 ± 1513
		DT	8555 ± 3341	9659 ± 3837	1104 ± 5108
Vertical Jump PV	m/s	NT	3.9 ± 0.4	4.1 ± 0.3	0.2 ± 0.2
		DT	3.5 ± 0.3	3.5 ± 0.2	0.0 ± 0.3
Vertical Jump PF*	N	NT	2978 ± 841	2730 ± 793	− 249 ± 386
		DT	3226 ± 1177	3672 ± 1208	445 ± 602
Vertical Jump AP	W	NT	1425 ± 339	1454 ± 337	28.2 ± 142.3
		DT	1235 ± 297	1277 ± 229	41.9 ± 121.3
Vertical Jump AV	m/s	NT	2.0 ± 0.2	2.0 ± 0.3	0.0 ± 0.2
		DT	1.6 ± 0.3	1.6 ± 0.3	0.0 ± 0.2

^a^indicates a significant main effect for time, and * indicates a significant group by time interaction

**Fig. 2:**
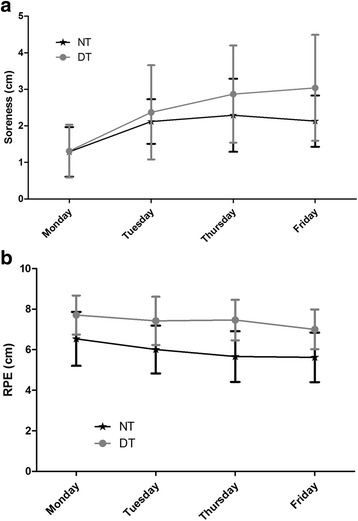
Weekly Averages for DOMS and RPE. **a** DOMS; **b** RPE. No significant differences were observed for the mean weekly rating of DOMS or RPE (*p* > 0.05). Data are presented as Mean ± SD

Significant group by time effects were observed for RPE (*p* < 0.05) but not DOMS (*p* > 0.05). RPE was significantly lower on the final 2 days (a mild tapering phase) of the training program versus like sessions in all previous weeks (Fig. 2). The NT group (1.8 ± 2.2 cm) rated the session as less strenuous than the DT group (3.8 ± 3.2 cm).

## Discussion

The results of the present investigation indicate that ingestion of supplemental casein protein at night or earlier in the day similarly influence adaptations to a resistance training program. Resistance training promotes significant increases in muscle strength and mass when the training stimuli is sufficient and protein intake is adequate relative to training [1–4]. In the current study, participants achieved an average training volume of 39,577 kg per week which provided the mechanical stimulus necessary for a significant increase in muscle strength and size. However, NT and DT groups did not differ in rates of change for these variables or measures of body fat and VJ performance.

Previously, it was reported that protein digestion and absorption occurs during the night just as it does during daytime hours or when most people are awake [7, 8]. One of the first studies to demonstrate the positive anabolic effect of exogenous nighttime protein on whole body protein balance studied older men who were provided 40 g of casein at 0200 via a nasogastric tube versus a volume matched bolus of water [7]. Furthermore, overnight muscle protein fractional synthetic rates were on average 55% greater with the provision of protein. The potential incremental impact of exercise on nighttime whole body and muscle protein balance was evaluated in young males engaged in an evening resistance exercise session and receiving 40 g casein, 30 min before bed (2330) [8]. Whole body protein synthetic rate and net protein balance were increased with the latter adjusted from a potentially net negative to positive balance (61 ± 5 vs − 11 ± 6 μmol/kg during 7.5 h of sleep) [8]. Furthermore, mixed muscle protein synthetic rates were ∼22% higher in a trial that included 40 g casein achieving a borderline level of significance (*p* = 0.05) [8].

Acute response studies (single dose) reinforce the idea that net whole body and muscle protein balance can be influenced by protein consumption and that the entire 24 h of a day is available for strategic protein consumption. Specifically, casein protein consumption during the NT period appears to be as responsible for the observed outcomes as casein protein consumption during the DT period. This capability permits greater flexibility during waking hours to achieve a positive protein balance. In theory, this translates to incremental increases in hypertrophy and strength over time. To challenge this concept, a recent study used males engaged in a progressive, 12-week resistance training program in the evening with one group consuming a protein-based supplement (27.5 g protein + 15 g carbohydrate) every night before sleep [6]. Significantly greater muscle strength as well as quadriceps CSA and microscopically greater increases in Type II muscle fiber size were observed in the protein supplemented group. Two important aspects of this experimental design are that the protein supplemented group consumed significantly more total protein daily (1.9 vs 1.3 g/kg) and the exercise stimulus was in the evening followed by low-protein meal (10 g protein, 37 g carbohydrate, 9 g fat), limiting possible conclusions about NT protein consumption opposed to total protein consumption. The current findings do not support localization to the DT period, favoring total daily protein intake as a predictor of adaptation when supplied in recommended quantities [10, 11]. Furthermore, supplements in the current study were consumed at least 3 h removed from exercise, preventing a timing effect relative to exercise that may have been present in the previous research.

Casein is an anecdotally obvious choice for NT protein supplementation, as it is not acid-stable and tends to gel in the stomach and empties in the small intestine at a slower rate than other proteins, resulting in a prolonged period of aminoacidemia [9, 14, 15]. Conceptually, this may be beneficial since sleep would prevent consumption of the next meal (e.g., breakfast) by about 6–8 h. While casein provides a steady release of amino acids for several hours, this may not only support muscle protein synthesis, but also help limit muscle protein breakdown [14]. When comparing whey to casein, whey leads to a faster and greater early hyperaminoacidemia and one potential compensating strategy would be to consume more casein. This could support maximizing MPS after high volume workouts [16]. The present study used 35 g of casein protein which is comparable to the level of casein used in previous nighttime protein studies [6–8]. Furthermore, nighttime calories, either in the form of protein or carbohydrate, did not lead to increases in fat mass. Most likely this is due to alignment between energy requirements and dietary provision; however, it also supports the notion that eating a smaller caloric load before sleep will not lead to changes in body composition versus ceasing to consume food or supplements past early evening. In a recent study, the provision of 54 g of protein before bed did not influence body composition [12]. The use of casein in the present study may also have influenced the results. Casein is digested over a period of ~ 6–7 h and may not robustly increase MPS when consumed in isolation compared to proteins like soy or whey [17]. The present study, limited by sample size, observed greater, albeit nonsignificant, advantages for DT vs. NT consumption of protein for increasing LST. Moreover, the sterile nature of the study (e.g., diet control, total protein control), in effect, limited total protein consumption due to the fact ~ 50 g of protein were supplemented daily and some quantities of lower quality plant proteins were consumed during participants efforts to meet carbohydrate intake requirements. Therefore, another hypothesis is that it could be possible for DT casein consumption to create an “elevated baseline” for hyperaminoacidemia, thereby reducing the absolute amount of dietary protein necessary to maximize protein synthesis in meals consumed during the 6–7 h postprandial period following casein supplementation [14]. As protein and its constituent amino acid, leucine, are believed to maximally stimulate protein synthesis at a certain threshold (e.g., 2.5-3 g leucine), it is conceivable that DT participants individual meals consumed up to 6–7 h following casein ingestion had a cumulative effect on amino acid and leucine concentrations, which was not possible with NT casein supplementation, and more frequently or robustly stimulated MPS [18, 19].

## Conclusions

The results of this study suggest that strategic, performance-supporting nutrition should be considered on a 24-h basis to optimize positive net body and muscle protein balance. Supplemental protein provided prior to sleep supports positive changes in muscle hypertrophy and strength in a manner like earlier in the day and in greater proximity to the timing of the training session. Therefore, NT consumption of protein may not be a special time period for protein consumption, but it may be treated as an equally effective time to consume protein to meet protein intake goals. One limitation of the study is the final number of participants. The rate of voluntary and guided dropouts was influenced by the naive state of the participants and long-term commitment to the rigors of the training schedule and nutrition compliance. It is possible that potential differences would be undetectable without more participants. These findings are helpful to coaches, dietitians, and athletes as they plan nutrition and supplementation strategies to support higher levels of strength training.
